# Blood Pressure Measurement Biases in Clinical Settings, Alabama, 2010–2011

**DOI:** 10.5888/pcd13.150348

**Published:** 2016-01-07

**Authors:** Keri Sewell, Jewell H. Halanych, Louise B. Russell, Susan J. Andreae, Andrea L. Cherrington, Michelle Y. Martin, Maria Pisu, Monika M. Safford

**Affiliations:** Author Affiliations: Keri Sewell, Jewell H. Halanych, Susan J. Andreae, Andrea L. Cherrington, Michelle Y. Martin, Maria Pisu, School of Medicine, Department of Medicine, University of Alabama at Birmingham, Birmingham, Alabama; Louise B. Russell, Institute for Health, Health Care Policy, and Aging Research and Department of Economics, Rutgers University, New Brunswick, New Jersey.

## Abstract

**Introduction:**

Blood pressure measurement in clinical care settings seldom follows the protocol recommended by national guidelines, potentially leading to overestimates or underestimates of blood pressure control. We evaluated blood pressure measurement methods as a source of bias in determining blood pressure control among community-dwelling adults with diabetes.

**Methods:**

In a community-based trial of patients with diabetes, we measured both “clinical blood pressure” (clinical BP) (taken by a community nurse or medical assistant instructed to “take the participant’s blood pressure like you do in your own clinic”) and “research blood pressure” (research BP) (research staff followed a guideline-concordant protocol). Each participant had both types of blood pressure assessment on the same day over the course of 2 hours.

**Results:**

The 227 participants had a mean age of 59 years; 86% were black and 74% were women. The mean clinical BP was 5 mm Hg higher than the mean research BP for systolic blood pressure (*P* < .001) and 2 mm Hg higher for diastolic blood pressure (*P* < .001). The proportion of participants whose clinical BP was 130/80 mm Hg or higher was 8 percentage points higher than the proportion whose research BP was 130/80 mm Hg or higher (*P* < .001), and the proportion whose clinical BP was 140/90 mm Hg or higher was 10 percentage points higher than the proportion whose research BP was 140/90 mm Hg or higher (*P* < .001). Among those aged 65 years or older, the proportion whose clinical BP was 130/80 mm Hg or higher was 10 percentage points higher than proportion whose research BP was 130/80 mm Hg or higher, and the proportion whose clinical BP was 140/90 mm Hg or higher was 14 percentage points higher than the proportion whose research BP was 140/90 mm Hg or higher. Whites and smokers had the greatest risk for having a clinical BP 5 mm Hg or more higher than their research BP.

**Conclusion:**

Measurement biases in clinical settings may be a component of observed poor blood pressure control rates in real-world settings.

## Introduction

High blood pressure is a cause of major illness and death in the United States. The seventh report of the Joint National Committee on Prevention, Detection, Evaluation, and Treatment of High Blood Pressure (JNC-7) established guidelines for the accurate measurement of blood pressure to aid in diagnosis and clinical management (1,2). However, blood pressure assessed in clinical settings may not always follow these guidelines, potentially resulting in inaccuracies that could affect clinical management. A 2002 National Institutes of Health (NIH) report observed that “many people think that an error of 5 mm Hg is insignificant, but this error at the 90–95 mm Hg range would miss the 21 million U.S. hypertensives. . . . Conversely, measuring blood pressure 5 mm Hg too high would falsely classify 27 million persons as having [hypertension]. At the cost of $1,000 per year to treat a patient, this would add $27 billion to the Nation’s health care bill to treat a nondisease” (3).

Some evidence supports the possibility that poor adherence to the JNC-7 guideline may lead to systematic blood pressure biases. In a study comparing specific equipment to usual approaches, patients with hypertension had 8.3 mm Hg higher readings when assessed using usual approaches, and 21% more were classified as having uncontrolled hypertension (4). Because the blood pressure was assessed using equipment that followed a JNC-7 concordant protocol but usual care had no protocol, the differences found in this study likely arose from a mix of equipment and protocol differences. Thus, this study was unable to identify the measurement error attributable to poor protocol alone. There is a lack of research focusing on the topic of measurement error in blood pressure measurement due to use of protocol.

We conducted a study designed to establish the average size and direction of measurement errors due to incorrect protocols for blood pressure measurement that might occur in routine clinical practice, and we sought to identify which groups would be most vulnerable to such errors. Because high blood pressure is more common in older people and because the Centers for Medicare and Medicaid Services has interest in optimizing quality and costs of care, we examined findings overall and by age (<65 years or ≥65 years). We used both the recommended diabetic blood pressure target of 130/80 mm Hg and a more broadly applicable target of 140/90 mm Hg in our analyses (5).

## Methods

### Study population

Data for this analysis were collected in conjunction with the Evaluating Community Peer Advisors and Diabetes Outcomes in Rural Alabama (ENCOURAGE) study, a group-randomized pragmatic trial of the effect of community peer advisors on diabetes outcomes. The details of recruitment and data collection are provided elsewhere (6,7). Briefly, participants were community-dwelling adults (≥19 years old) with diabetes who had a primary-care physician and were interested in improving their diabetes self-management. Baseline health assessments were conducted in community locations and included blood pressure measurement. The 227 people who had a clinical blood pressure (clinical BP) assessed before a research blood pressure (research BP) formed the study sample for this report. All participants provided informed consent, and the study was approved by the University of Alabama at Birmingham institutional review board.

### Blood pressure measurement

Blood pressure was measured during the biometrics assessment. Other data collected included height, weight, and waist circumference, and blood was collected by a finger stick. The atmosphere of the data collection area was therefore similar to a busy clinical setting.

Two methods of measuring blood pressure were used, and each participant received 1 measurement from each method. The clinical BP was assessed by registered nurses or medical assistants who worked at local clinics or medical facilities. They were instructed to “take the participant’s blood pressure like you do in your own clinic.” They were provided an automated LifeSource blood pressure monitor model UA-789 (A and D Medical), 3 blood pressure cuffs (medium, large, and extra-large), a chair, and a table on which participants could rest their arms. They were not trained in the research BP protocol (described below). A single BP reading was taken and recorded. Clinical BP and research BP assessments were done in physically separate areas, and staff performing these measures were asked not to interact to minimize contamination. Clinical BP staff was not formally monitored, but casual observation indicated that participants were generally seated with the arm resting on the table to obtain the blood pressure measurement.

In comparison, study staff were specifically trained in research BP measurement methods by using a protocol that closely followed JNC-7 recommendations (2) using the same model of automated blood pressure monitor as used for measuring the clinical BP. To minimize the influence of equipment biases, the monitors were not labeled, so a monitor could be used for research BP on one assessment day and for clinical BP on another day.

Before taking the research BP measurement, participants’ right arms were bared to the shoulder, and they stood with the right elbow bent 90 degrees at the elbow with the hand on the stomach. Arm length was measured from the acromion to the olecranon process using a Gulick II Plus anthropometric tape measure (G and S — Fibreflex, Inc). The midpoint of the arm was marked with an eyebrow pencil. The participant relaxed the arm, and the arm circumference was measured horizontally at the midpoint mark by using the tape measure (to ensure the tape measure was at the proper tension). The appropriate size blood pressure cuff was used based on the arm circumference (24–35.5 cm = medium cuff; 36–42 cm = large cuff; >42 cm = extra-large cuff).

The participant was then seated comfortably in a chair with back support and both feet flat on the floor. The participant’s brachial artery was marked with the eyebrow pencil. The cuff was placed snugly on the arm with the inflatable inner bladder centered over the brachial artery and the lower edge of the cuff about 1 inch above the natural crease of the elbow. After the cuff was properly placed, the participant was instructed to sit quietly without talking, eating, completing paperwork, or crossing his or her legs for 5 minutes. Research BP personnel used timers to ensure a full 5-minute rest. Blood pressure was then measured with the automated monitor and, after a 1-minute rest time, blood pressure measurement was repeated. The average of the 2 readings constituted the research BP.

All participants in this study had research BP assessed after the clinical BP, because we anticipated that measuring research BP before clinical BP would lead to similar levels of blood pressure and that the true biases between the methods would not be discernible. We anticipated that measurement order could affect results, as the effect of quietly resting in the research BP protocol could artificially impact the clinical BP measurement that followed. To confirm this suspicion, we assessed the research BP before the clinical BP in a separate 180 participants, and blood pressure levels were not significantly different.

### Analysis

Mean systolic and diastolic blood pressures obtained using clinical BP measurement and research BP measurement were compared using *t* tests. The proportions that were classified as having uncontrolled high blood pressure (≥140/90 mm Hg and, separately, ≥130/80 mm Hg) were compared by using the χ^2^ statistic. Participants were divided into 2 groups, one having an increase of 5 mm Hg or more in the clinical BP compared with the research BP and the other having less than 5 mm Hg increase in clinical BP compared with research BP. Participant characteristics including age, race, sex, and risk factors (smoking, high low-density lipoprotein cholesterol, insulin usage, and blood pressure medication usage) were compared in these 2 groups, using an unadjusted odds ratio. Variables with bivariate associations with *P* < .20 were included in a multivariable model predicting factors associated with having an increase of 5 mm Hg or more in the clinical BP compared with the research BP. All analyses were conducted using SAS v9.2 (SAS Institute Inc). Test results were considered significant at *P* < .05.

## Results

The study sample had 227 participants with diabetes (Table 1). Their mean age was 59 years; 86% were black and 74% were women. Thirty percent of participants were using 3 or more medications for their blood pressure, and 17% were not taking any medications for blood pressure. Forty-two percent were using insulin for their diabetes, and participants on average had had a diabetes diagnosis for 12 years.

**Table 1:** Characteristics of the Study Sample of Community-Dwelling Adults With Diabetes (n = 227), Overall and by Difference in Systolic Blood Pressure^a^, Alabama, 2010–2011

Characteristic^b^	All (N = 227), n (%)	Clinical BP <5 mm Hg Higher Than Research BP (n = 114), n (%)	Clinical BP ≥5 mm Hg Higher Than Research BP (n = 113), n (%)	*P* Value for Difference
Age <65 years	157 (69)	80 (51)	77 (49)	.74
Men	58 (26)	23 (40)	35 (60)	.07
Black	194 (86)	105 (54)	89 (46)	.003
Less than high school diploma	61 (27)	36 (59)	25 (41)	.11
Annual income <$20,000	106 (47)	58 (55)	48 (45)	.20
Current smoker	24 (11)	7 (29)	17 (71)	.03
On blood pressure medications	188 (83)	96 (51)	92 (49)	.58
On insulin	94 (42)	47 (50)	47 (50)	.95
Body mass index ≥30 kg/m^2^	164 (72)	84 (51)	80 (49)	.63
Hemoglobin A1c ≥7.0%	142 (64)	79 (56)	63 (44)	.03
Low-density lipoprotein cholesterol ≥100 mg/dL	133 (59)	68 (51)	65 (49)	.80

a For clinical blood pressure (clinical BP) measurement, nurses or medical assistants were instructed to “take the participant’s blood pressure like you do in your own clinic.” The research blood pressure (research BP) was measured following a protocol similar to Joint National Committee on Prevention, Detection, Evaluation, and Treatment of High Blood Pressure (JNC-7) recommendations (2).

b Missing value summary (presented as n with difference of < 5 mm Hg; n with difference ≥ 5 mm Hg): race, n = 2 (1;1); education, n = 2 (1;1); income, n = 28 (13;15); smoking status, n = 12 (4;8); insulin, n = 2 (1;1); hemoglobin A1c, n = 5 (3;2); low-density lipoprotein cholesterol, n = 1 (0;1).

**Figure 1:**
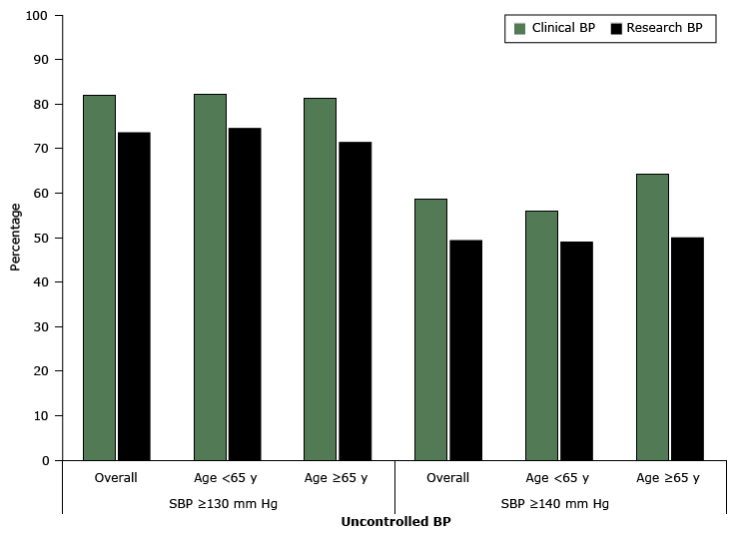
Percentage of participants with uncontrolled high blood pressure (mm Hg) as determined by clinical blood pressure (clinical BP) and research blood pressure (research BP), overall and by age group among community-dwelling adults with diabetes (n = 227), Alabama, 2010–2011. For clinical BP measurement, nurses or medical assistants were instructed to “take the participant’s blood pressure like you do in your own clinic.” The research BP was measured following a protocol similar to Joint National Committee on Prevention, Detection, Evaluation, and Treatment of High Blood Pressure (JNC-7) recommendations (2). Abbreviation: SBP, systolic BP. **Table:** 

**Figure 2:**
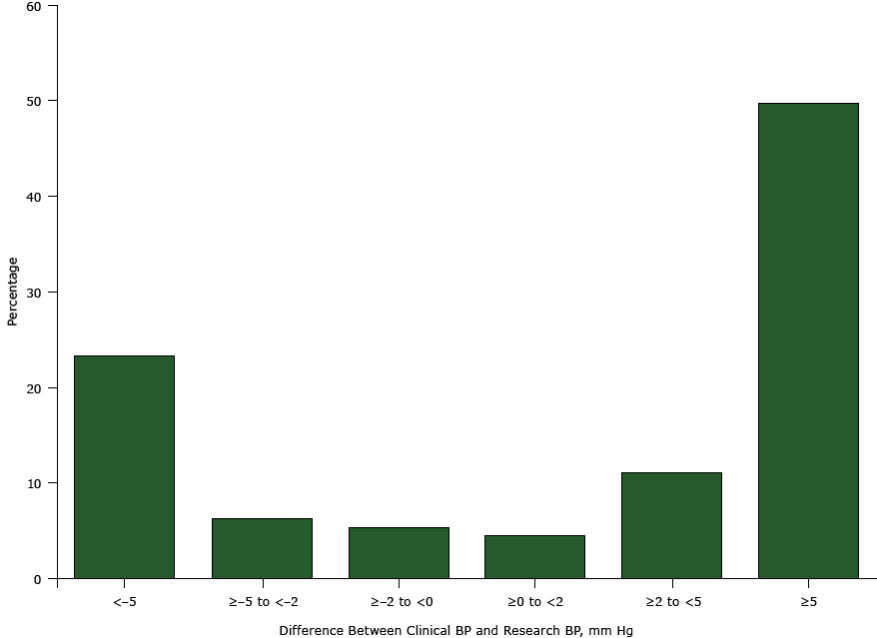
Differences between clinical blood pressure (clinical BP) and research blood pressure (research BP) among community-dwelling adults with diabetes (n = 227; data were missing for 3 participants), Alabama, 2010–2011. For clinical BP measurement, nurses or medical assistants were instructed to “take the participant’s blood pressure like you do in your own clinic.” The research BP was measured following a protocol similar to Joint National Committee on Prevention, Detection, Evaluation, and Treatment of High Blood Pressure (JNC-7) recommendations (2). **Table:** 

For systolic blood pressure, the mean clinical BP was 5.3 mm Hg higher than the mean research BP; for diastolic blood pressure, the mean was 2.1 mm Hg higher (both *P* < .001) (Table 2). On the basis of the recommended target of 130/80 mm Hg for people with diabetes, the proportion of participants classified as having uncontrolled high blood pressure was 8 percentage points higher when using clinical BP compared with research BP (82% vs 74%, *P* < .001) (Figure 1). On the basis of a recommended level of 140/90 mm Hg, the percentage of participants classified as having uncontrolled high blood pressure was 10 percentage points higher when using clinical BP compared with research BP (59% vs 49%, *P* < .001) (Figure 1). Half (50%, n = 113) of participants had a clinical BP that was 5 mm Hg or more higher their research BP (Figure 2).

**Table 2:** Mean Clinical Blood Pressure (Clinical BP) and Research Blood Pressure^a^ (Research BP) Among Adults With Diabetes, Overall and by Age Group, Among Community-Dwelling Adults With Diabetes, Alabama, 2010–2011

Age Group/Measurement	Clinical BP, Mean (SD)	Research BP, Mean (SD)	Difference	*P* Value
**All, N = 227**				
Systolic BP, mm Hg	140.3 (22.3)	135.0 (22.3)	5.3	<.001
Diastolic BP, mm Hg	85.6 (13.1)	83.5 (12.8)	2.1	<.001
**≥65 years, n = 70**				
Systolic BP, mm Hg	144.9 (20.6)	137.5 (20.7)	7.4	<.001
Diastolic BP, mm Hg	79.6 (10.6)	77.1 (10.4)	2.5	.013
**<65 years, n = 157**				
Systolic BP, mm Hg	138.2 (22.8)	133.9 (23.0)	4.3	<.001
Diastolic BP, mm Hg	88.2 (13.3)	86.3 (12.8)	1.9	.003

Abbreviation: SD, standard deviation.

a For clinical BP measurement, nurses or medical assistants were instructed to “take the participant’s blood pressure like you do in your own clinic.” The research BP was measured following a protocol similar to Joint National Committee on Prevention, Detection, Evaluation, and Treatment of High Blood Pressure (JNC-7) recommendations (2).

Blood Pressure	Clinical BP, %	Research BP, %
**SBP ≥130 mm Hg**		
Overall	81.9	73.6
Age <65	82.2	74.5
Age ≥65	81.4	71.4
**SBP ≥140 mm Hg**		
Overall	58.6	49.3
Age <65	56.0	49.0
Age ≥65	64.3	50.0

Difference Between Clinical BP and Research BP, mm Hg	Percentage
<−5	23.3
≥−5 to <−2	6.2
≥−2 to <0	5.3
≥0 to <2	4.4
≥2 to <5	11.0
≥5	49.8

We found that the difference between the clinical BP and research BP was consistent regardless of participant age group, and the difference was more pronounced in older participants. Seventy of our participants were aged 65 years or older, and in this subset, the mean systolic clinical BP was 7 mm Hg higher than the mean systolic research BP (145 mm Hg vs 138 mm Hg, *P* < .001), and the mean diastolic clinical BP was 3 mm Hg higher than the mean diastolic research BP (80 mm Hg vs 77 mm Hg, *P* = .01) (Table 2). The proportion of participants aged 65 years or older who were classified as having uncontrolled high blood pressure had clinical BP higher than research BP at both the 130/80 mm Hg cutoff (81% vs 71%, *P* < .001) and the 140/90 mm Hg cutoff (64% vs 50%, *P* = .001) (Figure 1).

Table 3 shows the results of the multivariable modeling to understand factors associated with having systolic clinical BP 5 mm Hg or more higher than research BP. Blacks had 71% lower odds than whites of having systolic clinical BP 5 mm Hg or more higher than research BP (odds ratio [OR], 0.29; 95% confidence interval [CI], 0.12–0.74). Those with hemoglobin A1c 7.0% or higher had 45% lower odds than those with hemoglobin A1c less than 7.0% (OR, 0.55; 95% CI, 0.30–1.00; *P* = .05). Current smokers had greater odds than nonsmokers (OR, 2.55; 95% CI, 0.96–6.75; *P* = .06). Men had higher odds than women, but this difference was not significant (OR, 1.62; 95% CI, 0.83–3.19; *P* = .16), and educational level was not significantly associated.

**Table 3:** Factors Associated With a Clinical Blood Pressure (Clinical BP) 5 mm Hg or More Higher Than Research Blood Pressure (Research BP)^a^ Among Community-Dwelling Adults With Diabetes, Alabama, 2010–2011

Characteristic	Unadjusted OR (95% CI)	*P* Value	Multivariable Adjusted OR^b^ (95% CI)	*P* Value
Age ≥65 vs <65 years	1.10 (0.63–1.93)	.74	NA	
Men vs women	1.77 (0.97–3.26)	.07	1.62 (0.83–3.19)	.16
Blacks vs whites	0.29 (0.13–0.69)	.005	0.29 (0.12–0.74)	.009
Less than high school diploma vs high school or more	0.61 (0.34–1.11)	.11	0.84 (0.44–1.64)	.61
Annual income <$20,000 vs all others	0.71 (0.42–1.20)	.20	NA	
Current smoker vs not current smoker	2.84 (1.13–7.17)	.03	2.55 (0.96–6.75)	.06
On any BP medications vs on none	0.82 (0.41–1.64)	.58	NA	
On insulin vs not	1.01 (0.60–1.72)	.95	NA	
BMI ≥30 kg/m^2^ vs <30 kg/m^2^	0.87 (0.48–1.55)	.63	NA	
Hemoglobin A1c ≥7.0% vs <7%	0.53 (0.30–0.93)	.03	0.55 (0.30–1.00)	.05
LDL ≥100 mg/dL vs <100 mg/dL	0.94 (0.55–1.59)	.80	NA	

Abbreviations: BMI, body mass index; CI, confidence interval; LDL, low density lipoprotein; NA, not included in the multivariable model; OR, odds ratio.

a For clinical BP measurement, nurses or medical assistants were instructed to “take the participant’s blood pressure like you do in your own clinic.” The research BP was measured following a protocol similar to Joint National Committee on Prevention, Detection, Evaluation, and Treatment of High Blood Pressure (JNC-7) recommendations (2).

b Variables with bivariate associations with *P* < .20 were included in multivariable model, which included 208 participants (19 dropped due to missing covariates).

## Discussion

In this study of 227 community-dwelling adults with diabetes, we found that blood pressure measured following typical approaches used in physician offices resulted in higher blood pressure assessments for most patients compared with measures obtained using the same equipment but closely following JNC-7 recommendations. We found that 8% to 10% of patients who would be considered to have uncontrolled high blood pressure by the clinical measurement actually had adequately controlled blood pressure by the research measurement. These results have implications for clinical management, quality assessments, performance-based reimbursement programs, and costs to patients and insurers.

Our findings suggest that common measurement approaches in real-world clinical settings tend to overestimate blood pressure, increasing the diagnosis of uncontrolled hypertension. Measurement biases in clinical settings may be a component of low rates of blood pressure control. We found that using incorrect protocols overestimated true systolic blood pressure levels on average by the same amount hypothesized in the 2002 NIH report (5 mm Hg) (3). Using the estimates in this report, improving blood pressure measurement techniques could result in US cost savings in the billions of dollars.

In addition to the effect on cost, reducing bias in blood pressure measurement may improve treatment burden, side effects, and quality of care measures. A recent study concluded that lowering systolic blood pressure below 120 mm Hg in diabetic patients did not improve cardiovascular outcomes and resulted in more adverse events, further emphasizing the importance of accurate blood pressure measurement (8).

Our results show that several groups are significantly more likely to have increased elevation of systolic blood pressure with clinical BP measures. Whites, current smokers, and those with controlled blood glucose were all significantly more likely to have 5 mm Hg or more higher systolic clinical BP compared with research BP. Men may also be more likely to have higher clinical BP compared with women. It is unclear why these groups were most affected by poor measurement technique, but each represents a group that could benefit from closer attention to blood pressure monitoring technique.

The implications of incorrect blood pressure assessment protocols may be most important for those aged 65 or older, among whom hypertension is most prevalent. In our study, the proportion of people aged 65 or older incorrectly classified as having uncontrolled high blood pressure was 10 to 14 percentage points higher, depending on the threshold considered. Inaccurate diagnosis of uncontrolled hypertension has substantial implications in the older population, including unnecessary polypharmacy and medication interactions, increased fall risk due to hypotension, side effects of medications, increased medication cost, and patients’ frustrations and sense of failing medical treatment (9).

Limitations to our study include its modest size in a mostly black sample with diabetes, which may limit generalizability. The local medical staff hired to measure clinical BP may not accurately represent other groups of medical staff with regard to blood pressure measurement technique and knowledge of the recommended measurement protocol. However, our study was able to show overestimation of uncontrolled high blood pressure even for the higher threshold of 140/80 mm Hg for people without diabetes. Furthermore, our population, composed of primarily minority participants with diabetes, is a high-risk group especially vulnerable to adverse cardiovascular events; thus, accurate blood pressure measurement and appropriate treatment are vital. Blacks in general are more susceptible to diabetes and hypertension and are an important study population. It is common in clinical settings to measure blood pressure with patients sitting on an examination table, with legs dangling and the arm unsupported. Our findings may therefore be conservative.

Because of our intentionally predominantly black study population, we did not have a large white population for comparison. Only 8 participants were both white and had a clinical BP that was less than 5 mm Hg higher than research BP. However, racial correlation with increased bias was still significant at *P* = .005. Future studies could help clarify the correlation strength and possible cause of why whites tend to be more affected by blood pressure measurement bias.

Methods used to assess blood pressure in typical clinical settings may overestimate true blood pressure levels that are assessed in concordance with JNC-7 recommendations, especially among older people. Groups such as whites and smokers may be at higher risk for this measurement bias, and these groups warrant future studies and attention. The magnitude of the overestimate is clinically important both for clinical management and quality assessment. Billions of dollars could potentially be saved annually, and patients could be spared adverse effects from medication with closer adherence to guideline-recommended approaches to blood pressure assessment.
